# Analysis of Social Media Impact on Opportunity Recognition. A Social Networks and Entrepreneurial Alertness Mixed Approach

**DOI:** 10.3390/e22030343

**Published:** 2020-03-17

**Authors:** Sebastian Ion Ceptureanu, Eduard Gabriel Ceptureanu, Marian Pompiliu Cristescu, Gurjeet Dhesi

**Affiliations:** 1 Department of Management, The Bucharest University of Economic Studies, 010374 Bucharest, Romania; eduard.ceptureanu@man.ase.ro; 2 Faculty of Economics, “Lucian Blaga” University of Sibiu, 550024 Sibiu, Romania; marian.cristescu@ulbsibiu.ro; 3 School of Business, London South Bank University, London SE1 0AA, UK; dhesig@lsbu.ac.uk

**Keywords:** social media, entrepreneurial opportunity recognition, entrepreneurial alertness, social networks

## Abstract

This paper investigates the effects of social media on entrepreneurial opportunity recognition. Combining the internal and external approaches of opportunity recognition, the study analyzes how social media influences the entrepreneurs in discovering new entrepreneurial opportunities. Structural equation modeling was used in this study, using the variance-based partial least squares (PLS)–structural equation modeling (SEM), on a sample of 354 entrepreneurs. We concluded that social media directly and positively influences entrepreneurial opportunity recognition while entrepreneurial alertness (internal approach) and social networks (external approach) partially mediates its indirect effects on entrepreneurial opportunity recognition. The study contributes to the existing literature by bringing new insights into the entrepreneurial opportunity recognition process by focusing on a poorly represented factor in the literature, social media.

## 1. Introduction

Entrepreneurship is intrinsically related to entrepreneurial opportunities identification and exploitation [1]. As such, entrepreneurial opportunity recognition is critical for entrepreneurs. Entrepreneurial opportunity involves two processes, recognition and exploitation [1,2,3,4], with various studies exploring the mechanisms entrepreneurs use to recognize them [5,6].

The entrepreneurs have to innovate or introduce new products or services and, therefore, are willing to search for new ideas. Consequently, they must gather, interpret and use information to recognize opportunities for their prospective businesses. In this vein, access to relevant information plays an important role in entrepreneurial opportunity recognition [7]. Various theories bring forward different determinants of entrepreneurial opportunity recognition process, with those emphasizing the personal characteristics of an entrepreneur, such as entrepreneurial alertness [8,9], and social networks [10] being dominant.

In the last decade, social media applications have dramatically changed the way entrepreneurs interact with stakeholders, other businesses and people. Entrepreneurs now use tools provided by social media, comprising technologies that allow the generation of user-created information and support user interaction [11], to interact with current and future customers [12], enabling the discovery of new customers and the collection of information [13]. The increasing use of social media applications makes social media a necessary platform for entrepreneurs to build up their own social networks [14]. Social media may enable entrepreneurial opportunities recognition by facilitating entrepreneurs’ interactions with peers. Similarly, the entrepreneurs take advantage of social media by initiating or strengthening their relationships with peers and prospective partners and customers [15,16].

However, it is questionable if social media influence entrepreneurial opportunity recognition in practice, since there are studies proving an inconsistent or even negative impact [9]. This, coupled with the very few existing studies on the topic, provided the rationale of this study. Therefore, the purpose of this paper is to investigate if and to what extent social media influences entrepreneurial opportunity recognition.

The paper is structured as follows: the first section, introduction, describes the rationale of the paper and the research questions; the second presents the literature review regarding entrepreneurial opportunity recognition and its relationship with entrepreneurial alertness, social networks and social media; the third section describes materials and methods used for the study; the fourth section presents the analysis and results achieved; finally, the last section discusses the findings and extensively presents the conclusions and future research avenues.

## 2. Literature Review and Hypothesis Development

### 2.1. Entrepreneurial Opportunity Recognition

Entrepreneurial opportunity recognition (EOR) involves finding a new means-end relationship that can be exploited for profit or potential value [1,3]. The literature provides two perspectives for understanding EOR [17]. The first, internally oriented perspective explores how the entrepreneurs’ internal knowledge, such as prior knowledge, creativity and cognitive processes, determines opportunity recognition [18,19,20,21]. The second, external perspective focuses on how entrepreneurs search for and acquire information and knowledge from external sources, especially through social networks [22].

Simultaneously, there are two, distinct approaches of the EOR process, one emphasizing the discovery while the second the creation of opportunities [23]. Entrepreneurial opportunity discovery is, according to the correspondent theory, an objective phenomenon, with opportunities existing independently of the entrepreneur [24]. Thus, their discovery implies active searching of the environment, with entrepreneurs focusing on changes in technology, consumer preferences and markets as sources of opportunities [25]. The entrepreneurs may use various IT applications, such as social media, to discover opportunities. In this paper, we are embracing this view, so from this point on opportunity recognition overlaps with opportunity discovery. The opportunity creation view is seen as emerging between the interactions of the entrepreneur and the environment [3]. It assumes that opportunity depends on the entrepreneur and is, therefore, a subjective phenomenon since entrepreneurs construct their opportunities [26,27,28]. By contrast, the creation view suggests that entrepreneurial opportunities do not exist but are created by entrepreneurs.

### 2.2. Entrepreneurial Alertness and Entrepreneurial Opportunity Recognition

The impact of Entrepreneurial alertness (EA) on the process of opportunity recognition is extensively researched [29]. EA enables entrepreneurs to better evaluate changes in the potential markets and, therefore, identify new opportunities, enhancing their ability to find new entrepreneurial opportunities, which, in turn, improves organizational performance and innovativeness. Simultaneously, it enables more complex scans for information from different areas to facilitate EOR [29]. In the same vein, entrepreneurs with higher levels of EA are more prone to recognize entrepreneurial opportunities by connecting ideas and knowledge [30]. EA emerges from environmental, market and technological changes [31], involving scanning and searching for new information [32]. Most entrepreneurs have a high level of EA during the process of EOR [33]. Various scholars [3,34] highlighted the positive role of EA in EOR, where EA increases the chances of initiating an entrepreneurial endeavor. Therefore, we proposed the following hypothesis:

**Hypothesis 1** **(H1).** *Entrepreneurial alertness has a positive and direct impact on entrepreneurial opportunity recognition.*

### 2.3. Social Networks and Entrepreneurial Opportunity Recognition

*Social networks* (SN) are considered important in EOR, especially in the early stages of venture initiation [35]. In various studies focused on EOR, SN was analyzed [9,10]. The existing studies on the topic usually focus on the strength of SN, on how users are connected and what impact they have on business survival [36]. Information is essential to the entrepreneurial process, enabling discovery and recognition of more opportunities by entrepreneurs [2,3,7]. Various peers (such as mentors, family, friends or casual acquaintances) may provide the entrepreneurs with information useful in recognizing entrepreneurial opportunities [7]. Moreover, high levels of SN provide advantages for entrepreneurs in terms of sharing specialized information and knowledge [37] and spiritual support, reduce knowledge gaps and uncertainties, increasing their confidence in identifying entrepreneurial opportunities [38,39]. Considerable evidence has shown that high-quality network relationships or superior network positions facilitate EOR [22,40]. Previous studies indicated that entrepreneurs have different skills to develop and manage SN [41] in which they can benefit in terms of EOR. As such, only some of the entrepreneurs met success. Therefore, we proposed the following hypothesis:

**Hypothesis 2** **(H2).** *Social networks have a positive and direct impact on entrepreneurial opportunity recognition process.*

### 2.4. Social Media and Entrepreneurial Opportunity Recognition

Social media (SM) comprises technologies that allow the generation of user-created information and support user interaction [11]. It allows the development of virtual relationships on many levels [42,43]. Various scholars demonstrated the use of SM to share information to find new ideas or find better opportunities [44,45]. At the same time, it seems that communication and information sharing is an important factor for SM use [46]. SM has modified the way entrepreneurs seek, search and gather information [47]. On social media platforms, entrepreneurs may find information regarding how to oversee their prospective businesses [48]. Recent studies have started to highlight the critical roles played by SM in fulfilling entrepreneurs’ information needs, such as knowledge exchange [49,50] or knowledge acquisition and sharing [51]. In addition, SM is useful in interacting with other entrepreneurs to solve problems [46,52]. Entrepreneurs are looking for advice on social media platforms from information providers, regardless if they are altruistic or not [44,45]. In addition, SM is used by entrepreneurs to enhance innovation and improve the productivity of their businesses [53,54]. SM allows entrepreneurs to maintain their existing contacts, make them more visible and facilitates intense interactions with peers, enabling EOR [43,55]. Finally, other studies demonstrate that SM facilitates the establishment of new ventures by supporting networking [45,52].

We assume that entrepreneurs, starting with limited knowledge and resources, are influenced by SM to sense trends and recognize entrepreneurial opportunities early on. Therefore, we proposed the following hypothesis:

**Hypothesis 3** **(H3).** *Social media have a positive and direct impact on entrepreneurial opportunity recognition.*

**Hypothesis 4** **(H4).** *Entrepreneurial alertness is mediating social media in the entrepreneurial opportunity recognition process.*

**Hypothesis 5** **(H5).** *Social networks (social media capability) is mediating social media in the entrepreneurial opportunity recognition process.*

**Figure 1:**
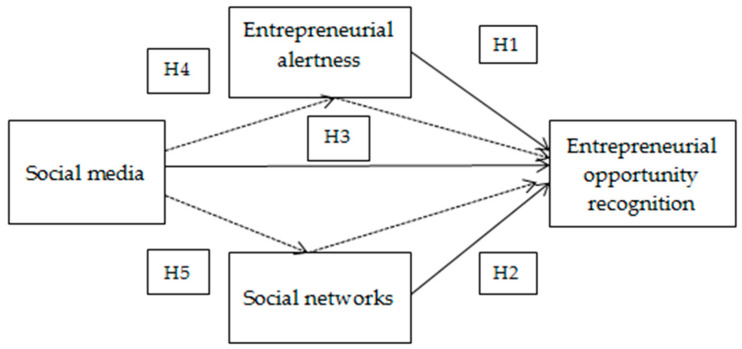
Conceptual model.

The conceptual model is presented below (Figure 1):

## 3. Materials and Methods 

### 3.1. Participants and Sampling Design

Considering the research subject, industries both rich in entrepreneurial opportunities and with a propensity for IT use were considered. In the end, IT-related creative industries’ companies (software NACE 5829; client-oriented software NACE 6201; IT consultancy NACE 6202; services for IT NACE 6209; and web portals NACE 6312) established in the Romanian capital, Bucharest, were selected as the research population. Out of 11,500 active companies in these industries, simple random sampling method calculations showed a minimum sample of 372 respondents.

In order to encompass all the dimensions underpinning the theoretical model, the authors have designed a questionnaire. It was developed as a Likert scale, 1 to 5, ranging from strongly disagree to strongly agree. The first version of the questionnaire was qualitatively and quantitatively tested during a focus group with 10 participants. The improved version was quantitatively tested on 30 entrepreneurs.

The questionnaires were distributed mainly via e-mail, with some delivered in person. In total, out of 400 questionnaires sent to entrepreneurs, representing 231 companies, out of which 358 were returned completed, with 4 rejected because of various errors and 354 valid for the analysis. The 231 companies’ database was developed during various research and European funded grants within The Bucharest University of Economic Studies. In the end, 206 SMEs provided the requested data. The sample structure is described in Table 1.

### 3.2. Instruments

The partial least squares (PLS) method was used [56] by calculating each variable variance explained by the principal construct [57]. The results show the variance of the common method bias does not constitute a problem. Structural equation modeling (SEM) was used, employing the variance-based PLS–SEM [58,59] and SmartPLS (2.0) software developed by SmartPLS GmbH, Germany.

Given that the scales were originally designed as measurements for studies in different settings, they were modified to suit the study context.

Entrepreneurial alertness construct was measured by using a previously developed scale [8,32], comprising items grouped in three processes: scanning and searching for information (8 items); association and connection (8 items); and evaluation and judgment (8 items), while Social networks construct was measured by using a proxy the entrepreneur’s network capability [23], comprising network building (4 items), network maintenance (3 items) and network coordination (4 items). Social media construct was measured by using a proxy, the social media capability, with 4 items [60,61]. The dependent variable, Entrepreneurial opportunity recognition construct, was measured by mixing three scales: [7,62,63]. In the end, 8 items were selected. All items are described in the Appendix A.

Demographic characteristics of the respondents described in the sampling section (age, gender and education) were used to control the possible adverse effects of these variables on EOR.

## 4. Analysis and Results

The cross-loadings were examined and the results are higher than the acceptable threshold (0.4) [64]. To measure reliability, Cronbach’s α and the composite reliability were used. The results are presented in Table 2 and demonstrate values higher than the 0.7 threshold value. Therefore, the constructs have a good degree of reliability. To test convergent validity, AVE (average variance extracted) was analyzed. With values above the 0.5 threshold, it proves a good degree of convergent validity. 

Table 3 discriminant validity. The results show good discriminant validity for the constructs.

To determine the extent and impact of constructs, the path coefficients were analyzed (Table 4).

We found a direct positive impact of Entrepreneurial alertness on Entrepreneurial opportunity recognition, confirming H1 hypothesis (*t*-value = 1.973, *p* < 0.05). In a similar vein, the H2 hypothesis is confirmed (*t*-value = 2.876, *p* < 0.01), proving a direct positive impact of Social networks on Entrepreneurial opportunity recognition. 

The results confirmed the H3 hypothesis, also (*t*-value = 3.259, *p* < 0.01), meaning Social media has a positive impact upon Entrepreneurial opportunity recognition.

The path coefficient for Social media and Entrepreneurial alertness shows that Entrepreneurial alertness is partially mediating Social media impact on Entrepreneurial opportunity recognition (*t*-value = 0.307, *p* < 0.001), confirming H4. 

Similarly, Social media and Social networks path coefficient show that Social media partially mediates the impact of Social networks on Entrepreneurial opportunity recognition (path coefficient = 0.316, *p* < 0.001), confirming H5.

The mediation effects of Entrepreneurial alertness and Social networks were assessed by the variance accounted (VA) [65]. The VAs (%) for constructs were close to 50 percent for each factor (49.76% and 48.96%, respectively), suggesting partial mediation (Table 5).

To evaluate the structural model, we used R^2^ (for the structural model’s goodness of fit), Q^2^ (for goodness of fit and the predictive power of the structural model) and *t*-values (for the validity of the hypotheses). The results show that all research hypotheses are confirmed (Table 6).

## 5. Discussion and Conclusions

This study was conducted with the aim of testing the effects of Social media on entrepreneurial opportunity recognition. The findings demonstrate once more that both the entrepreneurial alertness and social networks constructs positively influence entrepreneurial opportunity recognition, confirming other recent studies [8,9,10,29,35], while social media effects were found as being moderated by entrepreneurial alertness and social networks.

In terms of entrepreneurial alertness, from the three processes incorporated in the construct—scanning and searching for information; association and connection; evaluation and judgment—the first two mediates the most social media. As such, it supports the conclusions of other studies, non-related to entrepreneurial opportunity recognition, for scanning and searching [44,45,47,48] or association and connection [46,51,52]. With knowledge and information sharing instrumental in social media use [46], this, in turn, is reflected in Entrepreneurial alertness, since, according to social cognitive theory, knowledge is one of its determinants. With entrepreneurs requiring information, social media facilitates knowledge exchange [49] by following more users on social media platforms [50]. In addition, social media enables more complex searching and increase entrepreneurs’ interaction with other people on social media platforms [49,52]. Social media effects are also moderated by entrepreneurial alertness in relation to entrepreneurial opportunity recognition by improving entrepreneurs’ up-to-date on information, and staying aware of the latest trends in the markets.

Social media enhances the entrepreneur’s network capability, which in terms of social networks seems important. Entrepreneurs’ social competence and skills [66], proven as influencing entrepreneurial processes effectiveness, is expressed by social media. Social media can facilitate personal relationships between users but its strength resides in its capability to enable many-to-many interactions [11]. The study concludes that social media does exert influences on entrepreneurial opportunity recognition both independently and almost equally through social networks. Entrepreneurs are different in their abilities to build and manage their social networks, and these differences can result in variations in entrepreneurial opportunity recognition success [41]. For entrepreneurs, the use of social media surpasses the mere maintenance of personal connections. Nowadays they use it in creating, enlarging and strengthening networks [67], which, in turn, facilitate entrepreneurial opportunity recognition. This allows for networking and interaction with peers, entrepreneurs and potential customers from different locations, with similar or diverse entrepreneurial profiles [68]. In addition, networking trough social media is important in the entrepreneurial development process in terms of the effectuation process [68]. It allows entrepreneurs to increase social capital, facilitating identification and capitalization of opportunities and increasing the chances for entrepreneurial success [69].

This study also diverged from other research in that it examined whether social media effects are mediated by other, better established, factors for entrepreneurial opportunity recognition. For this study, entrepreneurial alertness and social networks factors were chosen and the results demonstrate that these factors moderate social media impact. This is an important contribution to the literature.

Still, the results should be placed in context. There are studies demonstrating that social media may actually hinder the entrepreneurs’ ability to recognize entrepreneurial opportunities because, paradoxically, it can determine a lack of socialization [9]. Peers or other entrepreneurs may not be eager to share relevant information and knowledge but only trivial ones. Lack of trust may be an issue, too. With untrustworthy or even inaccurate information offered by anonymous individuals, entrepreneurs using social media may encounter difficulties in identifying the real or most lucrative opportunities. Simultaneously, the huge volume of information available on social media determines more time and effort to sort it out and recognize the most appropriate opportunities.

In terms of research limitations, firstly, there is no differentiation in sample selection between early and well-established entrepreneurs. Social media impact may be different according to the business stage in a company’s lifecycle and this has to be further investigated [70]. 

Secondly, the industries selected for the study are abundant in entrepreneurial opportunities. A broader selection of industries may provide different results, depending on the entrepreneurs’ propensity to use social media in their entrepreneurial actions.

Thirdly, a more thorough insight into the specific applications and tools used by entrepreneurs to identify opportunities should be desirable. Perhaps, even a ranking of these social media tools, like in other research areas [71], even though their heterogeneity is high, may guide the entrepreneurs.

By including social media among the factors influencing entrepreneurial opportunity recognition, the entrepreneurs’ capacity to properly identify the critical factors is simultaneously enhanced and expanded. It may further motivate existing or prospective entrepreneurs to rely more on social media applications to increase entrepreneurial alertness and to develop social networks.

To conclude, this study examines the impact of social media on entrepreneurial opportunity recognition. It contributes to the existing literature by bringing new insights into the entrepreneurial opportunity recognition process by focusing on a poorly represented factor in the literature, social media.

## Figures and Tables

**Table 1:** Sample structure.

Firms’ Characteristics		%	Entrepreneurs’ Characteristics		%
NACE classification	NACE 5829	20.39%	Gender	Male	82.77%
	NACE 6201	23.79%		Female	17.23%
	NACE 6202	25.73%	Education *	ISCED 4 or less	0.56%
	NACE 6209	30.10%		ISCED 5 or 6	59.32%
Size (no. of employees)	Micro (< 10)	21.36%		ISCED 7 or more	40.11%
	Small (10–49)	35.44%	Age	Less than 35 years old	29.10%
	Medium (50–249)	43.20%		More than 35 years old	70.90%

* ISCED = International Standard Classification of Education. ISCED 4 or less is roughly equivalent up to post-secondary non-tertiary education. ISCED 5 and 6 are equivalent to short-cycle tertiary education and bachelor or equivalent. ISCED 7 or more represent master or doctoral.

**Table 2:** Reliability measures.

Constructs	Cronbach’s α	Composite Reliability	AVE
Social media (SM)	0.86	0.89	0.75
Entrepreneurial alertness (EA)	0.87	0.89	0.81
Social networks (SN)	0.85	0.88	0.79
Entrepreneurial opportunity recognition (EOR)	0.84	0.86	0.83

**Table 3:** Validity measures.

Constructs	SM	EA	SN	EOR
SM	0.867			
EA	0.852	0.902		
SN	0.846	0.837	0.886	
EOR	0.854	0.848	0.852	0.911

**Table 4:** Partial least squares (PLS) path model results.

Relation	*t*-Value	Path Coefficient
Age → EOR	0.123	−0.036
Gender → EOR	0.217	0.012
Education → EOR	0.282	0.027
EA → EOR	1.973 *	0.344
SN → EOR	2.876 **	0.331
SM → EOR	3.259 **	0.342
SM → EA	316.5 ***	0.307
SM → SN	328.1 ***	0.316

* *p* < 0.05; ** *p* < 0.01; *** *p* < 0.001.

**Table 5:** Mediating effects.

	EA				SN			
	Direct Effect	Indirect Effect	Total Effect	VA (%)	Direct Effect	Indirect Effect	Total Effect	VA (%)
SM → EOR	0.318	0.315	0.633	49.76	0.321	0.308	0.629	48.96

**Table 6:** Structural model criteria.

Constructs	R^2^	Q^2^
SM	–	–
EA	0.826	0.737
SN	0.809	0.712
EOR	0.817	0.741
Mean	0.818	0.730

## Appendix A

**Table A1:** Entrepreneurial alertness, Social networks, Social media and Entrepreneurial opportunity recognition constructs.

**Entrepreneurial Alertness**	
Scanning and search (8 items)	I have frequent interactions with clients and suppliers to acquire new information
	I always pay attention for new business ideas when looking for information
	I regularly read various publications to acquire new information
	I browse the Internet every day
	In my daily activities, I try to look for new business ideas
	I am an avid information seeker
	I am always actively looking for new information
	I regularly seek information from various information sources
Association and connection (8 items)	I often see new combinations of people, materials, or products
	I often make novel connections and perceive new or emergent relationships between various pieces of information
	I often find differences between the way I see certain situations and the way other people see them
	I often come up with new ideas and approaches to customer problems
	I often think “outside the box”
	I see links between seemingly unrelated pieces of information
	I am good at “connecting dots”
	I often see connections between previously unconnected domains of information
Evaluation and judgment (8 items)	“Seeing” potential new business opportunities comes very naturally to me
	I have a special alertness or sensitivity toward profitable opportunities
	I have a gut feeling for potential opportunities
	I can distinguish between profitable and not-so-profitable opportunities
	I have a good ability to sense profitable opportunities
	I have the ability to distinguish between high-value and low-value opportunities
	When it comes to business opportunities, I am good at filtering or blocking out insignificant information to make decisions
	When facing multiple opportunities, I am able to select the good ones
**Social Networks**	
Network building (4 items)	I am alert to market developments that create potential partnership opportunities
	I always encourage my business partners to introduce their peers to me
	I always look for opportunities to have lunches or dinners with prospective business partners
	I often invite prospective business partners to participate in various social activities
Network maintenance (3 items)	I can read others well and know how they are feeling in a given situation
	I know well about what others need and try to do what I can for them
	When I have disagreements with my partners, I usually strive to be flexible accommodate to reach a mutually satisfactory compromise
Network coordination (4 items)	I always analyzes what I would like to achieve with others
	I know well which business partners I can trust and whom I cannot
	I can well match my energy and resources to my different business partners
	I have a clear mind about the interdependence among my business partners
**Social Media**	
Social media (4 items)	I often use social media to obtain work-related information and knowledge
	I regularly use social media to maintain and strengthen communication with peers
	I can gain lots of knowledge by using social media
	I use social media to contact customers
**Entrepreneurial Opportunity Recognition**	
Entrepreneurial opportunity recognition (8 items)	I discover entrepreneurial opportunities in my activity
	I discover previously unnoticed entrepreneurial opportunities
	I am excited to search for unexploited entrepreneurial opportunities
	I constantly search for solutions to product issues that build on my experience
	I search for product related information that took the firm into existing product areas
	I like to discover new ways of doing things
	I prefer to find new uses for existing products
	I am a source of innovative ideas
